# Disability Identity Among Diverse Learners and Employees at an Academic Medical Center

**DOI:** 10.1001/jamanetworkopen.2022.41948

**Published:** 2022-11-10

**Authors:** Barbara Jerome, Magali Fassiotto, Jonathan Altamirano, Ken Sutha, Yvonne Maldonado, Peter Poullos

**Affiliations:** 1 Stanford University School of Medicine, Stanford, California

## Abstract

This survey study evaluates representation of persons with disabilities across demographic characteristics at an academic medical center.

## Introduction

Although 27% of US individuals have a disability,^1^ only 8% of workers with disabilities are employed in the health care industry^2^ compared with 14% of all US workers.^3^ Health care workers with disabilities have a unique understanding of the challenges patients with disabilities may experience and are therefore critical to reducing health disparities.^4^ Increasing the representation of persons with disabilities (PWDs) in health care is essential to ensuring workforce equity. This survey study sought to understand the representation of PWDs in an academic medical center across diverse demographic characteristics. This understanding may aid in increasing the representation of PWDs to improve patient care through innovations in clinical care, research, and medical education.

## Methods

Between November and December 2020, faculty, students, trainees, and staff across Stanford Medicine, which encompasses the School of Medicine, Stanford Health Care, and Stanford Children’s Health, participated anonymously in a survey on institutional justice and equity. The survey included 2 questions about disability status, one asking if respondents had a qualifying condition by the Americans with Disabilities Act (ADA) definition^5^ and another probing their sense of personal identity. The study was deemed exempt by the Stanford University School of Medicine institutional review board, with a waiver of informed consent, because the study was non–human participant research. This study followed the STROBE and AAPOR reporting guidelines.

Disability status by demographic characteristics are reported. Descriptive statistics were calculated and χ^2^ tests conducted in SAS, version 9.4 to analyze quantitative responses. Significance was defined at 2-sided *P* < .05.

## Results

Of an estimated 28 800 individuals offered the survey, 3311 responded. Of these respondents, 874 (26.4%) reported having an ADA-defined disability; 249 of these respondents (28.5%) self-identified as disabled. Faculty were less likely to identify as disabled compared with staff (25/514 [4.9%] vs 167/1926 [8.7%]; *P* = .004). Those who identified as transgender, did not identify with a binary gender, or declined to report their gender identity were twice as likely as cisgender respondents to identify as having a disability (23/148 [15.5%] vs 244/3163 [8.2%]; *P < *.001). Those who identified as sexual minority individuals (lesbian, gay, homosexual, bisexual, or other) were more than twice as likely as heterosexual/straight individuals to identify as having a disability (65/384 [16.9%] vs 194/2805 [6.9%]; *P* < .001). A greater proportion of sexual minority individuals than heterosexual/straight individuals who reported an ADA-defined disability did not identify as disabled (103/384 [26.8%] vs 509/2805 [18.1%]; *P* = .005). Asian respondents were less likely than White respondents to identify as disabled (37/746 [5.0%] vs 146/1680 [8.7%]; *P* < .001) (Table).

**Table.** Disability by Demographic Characteristics

Characteristic	Total sample, No. (%) (N = 3311)	Self-identified as disabled, No. (%) (n = 267)^a^^,^^b^	*P* value	ADA-defined disability but did not self-identify as disabled, No. (%) (n = 625)^a^^,^^c^	*P* value
Role					
School of medicine faculty	514 (16.2)	25 (4.9)	.004	99 (19.3)	.67
Staff^d^^,^^e^	1926 (60.6)	167 (8.7)	NA	355 (18.4)	NA
Staff management^f^	324 (10.2)	22 (6.8)	.26	71 (21.9)	.14
Students and trainees^g^	415 (13.0)	40 (9.6)	.53	81 (19.5)	.61
Graduate, medical, or PA students	225 (7.0)	NA	NA	NA	NA
Postdoctoral scholars	136 (4.3)	NA	NA	NA	NA
Residents	54 (1.7)	NA	NA	NA	NA
Missing^h^	132 (4.0)	13 (9.8)	NA	19 (14.4)	NA
Gender^i^					
Cisgender					
Man^d^	846 (25.6)	55 (6.5)	NA	149 (17.6)	NA
Woman^d^	2317 (70.0)	189 (8.2)	NA	447 (19.3)	NA
Other, declined, or no answer^j^	148 (4.5)	23 (15.5)	<.001	29 (19.6)	.02
Sexual orientation					
Sexual minority^k^	384 (11.6)	65 (16.9)	<.001	103 (26.8)	.005
Bisexual	133 (4.0)	NA	NA	NA	NA
Lesbian, gay, or homosexual	190 (5.7)	NA	NA	NA	NA
Other^l^	61 (1.8)	NA	NA	NA	NA
Straight/heterosexual^d^	2805 (84.7)	194 (6.9)	NA	509 (18.1)	NA
Declined or no answer	122 (3.7)	8 (6.6)	NA	13 (10.7)	NA
Race and ethnicity					
Asian	746 (22.5)	37 (5.0)	.001	101 (13.5)	.72
URM^m^	686 (20.7)	64 (9.3)	.88	124 (18.1)	.14
American Indian/Alaska Native	8 (0.2)	NA	NA	NA	NA
Black/African American	155 (4.7)	9 (5.8)	NA	27 (17.4)	NA
Hispanic/Latinx	406 (12.3)	40 (9.9)	NA	69 (17.0)	NA
Native Hawaiian/other Pacific Islander	36 (1.1)	NA	NA	NA	NA
≥2 URM races and/or ethnicities	81 (2)	NA	NA	NA	NA
White^d^	1680 (50.7)	146 (8.7)	NA	369 (22.0)	NA
Other^l^	199 (6.0)	20 (10.1)	NA	31 (15.6)	NA

Abbreviations: ADA, Americans with Disabilities Act; NA, not applicable; PA, physician assistant; URM, underrepresented in medicine.

^a^ Row percentages are shown.

^b^ Participants were asked: “Do you identify as a disabled individual and/or a person with a disability? This question specifically refers to your sense of personal identity.” Participants who did not report an ADA-defined disability could choose to identify as a person with a disability.

^c^ Participants were asked: “By the [ADA] definition, do you have a medical condition that would qualify as a disability?” Participants were given the definition from the ADA,^5^ provided the following list of conditions that would be considered a disability, and made aware that this list is not intended to be exhaustive: deafness or other hearing loss, HIV infection, blindness or low vision, multiple sclerosis, diabetes, muscular dystrophy, cancer, major depressive disorder, epilepsy, bipolar disorder, intellectual disability, posttraumatic stress disorder, partial or completely missing limbs, obsessive-compulsive disorder, autism, schizophrenia, attention-deficit disorder, mobility limitations, attention-deficit/hyperactivity disorder, cerebral palsy, dyslexia, speech and language disabilities, other learning disabilities, chronic illness, orthopedic or rheumatologic conditions, and autoimmune disorders.

^d^ Reference category for χ^2^ tests.

^e^ Administrative, medical (nonphysician), research, and service staff at Stanford Medicine.

^f^ Respondents from Stanford Health Care and Stanford Children’s Health; respondents had the option to self-report their roles as medical or nonmedical management.

^g^ Cross-tabulations are reported in aggregate for students and trainees to maintain the confidentiality of respondents given small cell sizes.

^h^ Respondents who did not choose the Stanford University School of Medicine, Stanford Health Care, or Stanford Children’s Health as their institution were not asked to report their role and were excluded from χ^2^ tests.

^i^ Cisgender man and woman categories were collapsed into a single category for statistical tests. Gender identities are reported in mutually exclusive categories, although respondents were allowed to select more than 1 identity.

^j^ Respondents who identified as transgender, gender nonconforming, gender nonbinary, genderqueer, or other; those who preferred not to disclose or chose not to answer; and any combination of categories, including woman and man. Responses for individual categories are not reported to maintain the confidentiality of respondents given small cell sizes.

^k^ Respondents who selected lesbian, gay, homosexual, bisexual, or other as sexual orientation. Cross-tabulations between these categories and disability categories are not reported to maintain the confidentiality of respondents given small cell sizes.

^l^ Other given in the survey question.

^m^ Respondents who selected American Indian/Alaska Native, Black/African American, Hispanic/Latinx, and/or Native Hawaiian/other Pacific Islander. Cross-tabulations between some of these categories and disability categories are not reported to maintain the confidentiality of respondents given small cell sizes.

## Discussion

The prevalence of self-reported disability at Stanford Medicine found in this study was similar to the national prevalence (27%),^1^ but only 28.4% of respondents who reported an ADA-defined disability personally identified as disabled. Faculty may have been less likely than staff to embrace disability due to internalized ableism and misconceptions surrounding the productivity of PWDs. Sexual minority individuals with an ADA-defined disability and Asian individuals may have been less likely to identify as disabled due to fears of facing more discrimination from having multiple intersecting marginalized identities.^6^

This study is limited by self-selection bias as individuals who responded to the survey may be more likely to self-identify as disabled. Given that the survey was distributed through anonymous listservs for the broadest reach and to ensure confidentiality, we also cannot report a response rate.

Further research should investigate whether disability identity and having multiple marginalized identities affects the likelihood of seeking and access to accommodations. Ensuring that health care workplaces are accessible is necessary to promote an inclusive environment for PWDs.^4^
